# NOACs vs. Warfarin for Stroke Prevention in Nonvalvular Atrial Fibrillation

**DOI:** 10.7759/cureus.1395

**Published:** 2017-06-26

**Authors:** Priya Patel, Jheel Pandya, Madeline Goldberg

**Affiliations:** 1 University of Central Florida College of Medicine

**Keywords:** warfarin, atrial fibrillation, novel oral anticoagulant, stroke, ischemic stroke, vitamin k antagonist, rivaroxaban, apixaban, edoxaban, dabigatran

## Abstract

Atrial fibrillation is a heart arrhythmia associated with increased risk for ischemic stroke. Vitamin K antagonists were developed to decrease a patient's clotting risk; however, these medications require therapeutic monitoring and have several drug interactions. Novel oral anticoagulants (NOACs) were developed as an alternative to vitamin K antagonists and several studies have evaluated the ability of NOAC to decrease clotting as well as the risk of major bleeding in comparison to vitamin K antagonists, such as warfarin. This study has found that NOACs are as effective as warfarin in reducing stroke and systemic embolism through anticoagulation. Notably, NOACs have a decreased risk of significant bleeding and other secondary adverse events.

## Introduction and background

Atrial fibrillation (AF) is the most common type of cardiac arrhythmia. It affects an estimated 2.7 to 6.1 million Americans and is associated with significant morbidity and mortality because of its potential to cause stroke and other thromboembolic events [1]. The risk of stroke or other thromboembolic events is increased five-fold, with AF accounting for over 15% of all strokes in the United States (US); therefore, anticoagulation is recommended for stroke prevention in patients with AF [2]. Vitamin K antagonists were the only type of oral anticoagulant approved for stroke prevention until 2008 [3]. While these drugs provide optimal anticoagulation and are effective for the prevention of thromboembolism, they require regular monitoring and have several drug and food interactions, resulting in poor compliance [4].

Since 2008, several novel oral anticoagulants (NOACs) have been introduced to the market in the European Union and the US based on their efficacy, safety, and noninferiority to warfarin [3]. These anticoagulants include either factor Xa inhibitors or direct thrombin inhibitors and are much more expensive than warfarin. The NOACs have several benefits over the vitamin K antagonists. They do not require routine blood testing for international normalized ratio (INR) monitoring because they are administered at a fixed daily dose, have much fewer drug interactions, and have a rapid onset and offset of action with a wide therapeutic window [5]. In urgent situations, the effects of warfarin can be reversed using prothrombin complex concentrate, fresh frozen plasma, and vitamin K while there is no reversal agent available for NOACs. Adverse events, especially the risk of bleeding associated with NOACs in comparison to warfarin, are also an issue. This brings up the question of whether NOACs or warfarin should be the drug of choice for stroke prevention in AF. Based on current evidence, this article seeks to answer the following question: In patients with AF, are NOACs more efficacious than warfarin in preventing stroke or other thromboembolic events?

## Review

### Novel Oral Anticoagulants

To gain a better understanding of the effectiveness of novel oral anticoagulants (NOACs) and of any adverse events related to NOACs versus warfarin for stroke or systemic emboli prevention, multiple systematic reviews have been performed. A systematic review published in 2013 by Bruins, Slot, and Berge included 10 randomized, controlled trials from 1950-2013 using different sources like Cochrane Library, MEDLINE, EMBASE, Stroke Trials Directory, Clinical Trials, Current Controlled Trials, and Google Scholar and through information provided by pharmaceutical companies and authors of pertinent published trials. The review included data from 42,084 participants with a confirmed diagnosis of atrial fibrillation (AF) or atrial flutter randomized into groups of either dose-adjusted warfarin (INR 2.0-3.0) and any Xa inhibitor (apixaban, betrixaban, edoxaban, idraparinux, or rivaroxaban). Of the 10 trials, 4 were double-blinded, 5 partially blinded, and 1 was open labeled. Primary efficacy endpoints included stroke (ischemic and hemorrhagic) and systemic embolism (SE) with a focus on the adverse effect of bleeding. Based on the analysis of the 10 trials, the overall odds ratio was 0.78 (95% confidence interval (CI), 0.69-0.89), indicating that there was a statistically significant decrease in stroke or SE with factor Xa inhibitors compared to warfarin in patients with AF. With regards to adverse events, a significant reduction in major bleeding (hemoglobin drop >> 2 episodes) was observed in the factor Xa inhibitor group with an odds ratio of 0.89 (95% CI, 0.81-0.98) [6].

Another meta-analysis with a greater number of participants was performed to compare NOACs with warfarin for the prevention of stroke and embolic events in patients with AF. Hicks et al. reviewed abstracts and data using different sources, such as Medline, EMBASE, and grey literature searches. A total of 12 studies with a total population of 77,011 participants were analyzed for all stroke and SE outcomes as well as bleeding outcomes [7]. Overall, NOACs demonstrated a reduction in stroke or SE compared to warfarin (odds ratio (OR) 0.85, 95% CI, 0.75 to 0.98) and a 14% reduction in mortality (OR 0.86, 95% CI 0.82 to 0.91). In this study, they also analyzed major bleeding and increase in stroke or systemic embolic events after the 30-day end-of-study switch to warfarin. There was an increased risk of stroke or embolism (OR 2.60, 95% CI, 1.61 to 4.18) and increase in major bleeding (OR 2.19, 95% CI, 1.42 to 3.36). Generally, NOACs demonstrated superiority to warfarin in preventing stroke or SE as well as mortality in patients with AF. The post-study switch demonstrated an increase in the risk of stroke and bleeding after switching back to warfarin, which is significant to consider in clinical practice [7].

### Rivaroxaban

The ROCKET AF was a large, global (45 countries), randomized, double-blinded, and double-dummy trial. A total of 14,264 patients were randomized to either the rivaroxaban group (N=7,131) or the warfarin group (N=7,133). Both groups had similar characteristics with a median age of 73 years, 39.7% female, median blood pressure of 130/80, mean and median CHADS2 score of 3.5 and 3.0, and coexisting illnesses: 55% with previous stroke/SE/transient ischemic attack, 62% with congestive heart failure, 90% with hypertension, 40% with diabetes mellitus, and 17% with previous myocardial infarction. The types of AF within the group were also similar: 81% with persistent AF, 17.5% with paroxysmal AF, and 1.4% with new onset AF [8].

The trial included patients with nonvalvular atrial fibrillation (NVAF) documented by electrocardiography (ECG) with moderate-high risk for stroke based on a CHADS2 score of 2 or more. The rivaroxaban group received a daily dose of 20 mg or 15 mg compared to the warfarin group, which received an adjusted warfarin dose to maintain an INR of 2-3. The therapeutic range for an INR of 2.0-3.0 was maintained at a mean of 55% all the time. The primary outcome for the trial included stroke (ischemic or hemorrhagic) and SE usually established through neuro-imaging or angiography. Within the per-protocol population (included in the primary analysis), stroke or SE occurred in 188 patients in the rivaroxaban group (1.7% per year) and in 241 patients in the warfarin group (2.2% per year) with a hazard ratio of 0.79, 95% CI, 0.65-0.95, P<0.001. Based on the statistical analysis and the reduction in stroke or SE, the trial concluded that rivaroxaban was comparable to warfarin. There is a serious complication of bleeding with anticoagulation and the incidence of fatal or critical bleeding was less in the rivaroxaban group but the frequency of bleeding from gastrointestinal sites was more frequent in the rivaroxaban group. Overall, the trial showed a decrease in stroke and critical bleeding in rivaroxaban compared to warfarin, with several advantages to using Xa inhibitors, such as fewer drug interactions, rapid onset/offset, and no requirement for regular INR monitoring [8].

In a separate study, J-ROCKET AF, a randomized, double-blinded, and double-dummy trial, focused specifically on the Japanese population. Of the 1,280 randomized patients with NVAF, 1,274 were included in the per-protocol population and were separated into the rivaroxaban group (n=639) receiving 15 mg daily and the warfarin group (n=639) with an adjusted dose to maintain an INR of 2.0-3.0. The baseline characteristics for both groups were similar with a mean age of 71.1 years, mean CHADS2 score of 3.25, 19.4% female, and coexisting illness: 40.8% congestive heart failure, 79.5% hypertension, 38% diabetes mellitus, and 7.7% prior myocardial infarction [9].

In the J-ROCKET AF trial, the primary endpoint of stroke or SE occurred in 11 patients in the rivaroxaban group (1.26% per year) and in 22 patients in the warfarin group (2.61% per year) with a hazard ratio of 0.49, 95% CI, 0.24-1.00, P-0.50. For the primary endpoint, the absolute risk reduction (ARR) = 1.72%, relative risk reduction=49%, and number needed to treat = 58. The total risk of bleeding associated with anticoagulation was higher in the rivaroxaban group at 18.04% per year compared to the warfarin group at 16.42% (hazard ratio 1.11; 95% CI, 0.87-1.42); however, the risk of major bleeding was decreased in the rivaroxaban group at 3.00% per year compared with 3.59% in the warfarin group and had a hazard ratio of 0.85 (95% CI, 0.50-1.43). Based on the primary endpoint (stroke or SE) and the adverse events (bleeding), the study concluded that rivaroxaban was comparable to warfarin for stroke and SE prevention in AF patients [9].

### Direct Thrombin Inhibitors

A generalized review was done to compare direct thrombin inhibitors (DTIs) to vitamin K antagonists in Lima, Peru. This systematic review searched various databases, such as Cochrane Stroke Group Trials Register, Cochrane Central Register of Controlled Trials, MEDLINE, EMBASE, and LILACS. Three review authors performed data extraction and risk-of-bias assessments, where they compared all DTIs combined with warfarin. Overall, eight studies were included, consisting of 26,601 participants that were involved in the primary analysis. Results demonstrated that the odds of vascular death and ischemic events were not significantly different between all DTIs and warfarin (OR 0.94, 95% CI, 0.85-1.05). However, 150 mg twice daily of dabigatran was superior to warfarin with a borderline statistically significant effect (OR 0.86, 95% CI, 0.75 to 0.99). Major bleeding events, such as hemorrhagic strokes, were less frequent with DTIs (OR 0.87, 95% CI, 0.78 to 0.97), but adverse events leading to the discontinuation of DTIs were more frequent [10].

In 2009, another trial examining dabigatran found that dabigatran at 110 mg lowers the risk of stroke with less risk of major bleeding. In this trial, patients with AF were randomly assigned to receive either warfarin or dabigatran (at low or high dose - 110 vs. 150 mg, respectively). All patients had AF documented on electrocardiography at the time of screening or within the next six months. Researchers examined the risk of stroke or SE and found a statistically significant decreased incidence of stroke in the 110 mg dabigatran group compared with the warfarin group (1.53% vs. 1.69% per year, respectively). The relative risk with dabigatran was 0.91 with a 95% CI of 0.53-0.82, P<0.001. With reference to the bleeding risk, no statistically significant difference was discovered between patients taking dabigatran, both high or low dose, and warfarin. The rate of hemorrhagic stroke was significantly lower in the group taking 110 or 150 mg of dabigatran vs. warfarin (p<0.001). This finding correlates with the study’s conclusion that dabigatran at either the high dose (150 mg) or the low dose (110 mg) has a comparable ability to lower the stroke risk of patients, with a decreased risk of major bleeding events [11].

### Apixaban

A particular NOAC, apixaban, was compared to warfarin in a randomized, double-blind trial with 18,201 patients with AF and at least one risk factor for stroke. Apixaban was given at 5 mg twice daily in comparison to warfarin, with the primary outcome being ischemic or hemorrhagic stroke or SE. Secondary outcomes were death from any cause and myocardial infarction, and the primary safety outcome was major bleeding [12].

Patients in the apixaban group had a lower primary outcome rate (stroke or SE) at 1.27% compared to those in the warfarin group at 1.60% (hazard ratio with apixaban, 0.79; 95% CI, 0.66 to 0.95), and were statistically significant for noninferiority and superiority. The primary safety outcome of major bleeding was 2.13% per year in the apixaban group compared with 3.09% in the warfarin group (hazard ratio (HR) 0.69, 95% CI, 0.60 to 0.80). Death from any cause was borderline significant at 3.52% with apixaban and 3.94% with warfarin (HR 0.89, 95% CI, 0.80 to 0.99). Overall, apixaban proved to be statistically significantly superior to warfarin in preventing stroke or SE while also causing less major bleeding and death from any cause [12].

### Edoxaban

Edoxaban, another factor Xa inhibitor, was studied in phase III trials and compared with warfarin. This multicenter study was double-blinded with respect to the factor Xa inhibitor, edoxaban, and single-blinded with respect to warfarin. In this study, 536 patients were randomly assigned to either warfarin or a low, medium, or high dose of 30, 45, or 60 mg of edoxaban a day. This three-month trial measured stroke prevention as a prior outcome and bleeding risk as an adverse event. The trial found a 20.3% lower risk of adverse bleeding events in patients taking 30 mg edoxaban instead of warfarin daily. The lowest dose of edoxaban, 30 mg, had the lowest risk of major bleeding; however, the bleeding risk was comparable to that with warfarin and other doses.of edoxaban. Investigators cautioned that patient weight should be considered when prescribing edoxaban as patient weight <60 kg was associated with increased risk of bleeding events. The strengths of this study include a preintervention group analysis that found no significant baseline differences between participants randomly assigned to either a warfarin or an edoxaban (30, 40, or 60 mg) daily dose [13].

### Rivaroxaban, Dabigatran, Apixaban

A large-scale, recent, retrospective observational study, published in 2016, examined 43, 299 patients with NVAF, residing in Denmark, between 2011 and 2015. P-values <0.05 were considered statistically significant. The absolute risk of stroke or thromboembolism for warfarin was 2.01% (95% CI, 1.80-2.21). Compared to warfarin, the absolute risk differences of dabigatran were 0.11% (95% CI, -0.16 to 0.42); rivaroxaban 0.05% (-0.33 to 0.48), and apixaban 0.45% (-0.001 to 0.93). Regarding the adverse effect of intracranial bleeding, the absolute risk for warfarin was 0.60 (0.49 to 0.72) with the absolute risk differences of dabigatran at -0.34% (-0.47 to -0.21), -0.13% (-0.33 to 0.08) for rivaroxaban, and -0.20% (-0.38 to -0.01) for apixaban. Based on this data, this study found no statistically increased risk of stroke between NOACs (dabigatran, rivaroxaban, and apixaban) and warfarin, and a significantly decreased risk of intracranial bleeding in the dabigatran and apixaban groups [14].

## Conclusions

Most studies have demonstrated a statistically significant reduction in risk for strokes or embolisms with the use of NOACs, while few have shown them to be equally efficacious to warfarin. Overall, NOACs are superior to warfarin based on their efficacy for ischemic stroke prevention in patients with NVAF, reduced number of major bleeding events, and convenience of usage. In clinical practice, warfarin compliance is challenging for patients because regular checks of INR are needed during therapy to avoid the increased risk of bleeding. With NOACs, regular checks are not necessary, potentially resulting in increased patient compliance. Patient compliance with NOACs (estimate $400) could suffer because of their excessive cost compared to warfarin (estimate $4). Research is still lacking to establish if one NOAC is superior to another. 
